# Calcium-stimulated calcitonin - The “new standard” in the diagnosis of thyroid C-cell disease - clinically relevant gender-specific cut-off levels for an “old test”

**DOI:** 10.11613/BM.2018.030710

**Published:** 2018-10-15

**Authors:** Martin B. Niederle, Christian Scheuba, Alois Gessl, Shuren Li, Oskar Koperek, Christian Bieglmayer, Philipp Riss, Andreas Selberherr, Bruno Niederle

**Affiliations:** 1 Division of General Surgery, Department of Surgery, Medical University of Vienna, Vienna, Austria; 2 Division of General Anesthesia and Intensive Care Medicine, Department of Anesthesia, Intensive Care Medicine and Pain Medicine, Medical University of Vienna, Vienna, Austria; 3 Division of Endocrinology and Metabolism, Third Department of Internal Medicine, Medical University of Vienna, Vienna, Austria; 4 Division of Nuclear Medicine, Department of Biomedical Imaging and Image-Guided Therapy, Medical University of Vienna, Vienna, Austria; 5 Department of Pathology, Medical University of Vienna, Vienna, Austria; 6 Clinical Institute for Medical and Chemical Laboratory Diagnostics, Medical University of Vienna, Vienna, Austria; 7 Former Chief of the Section “Endocrine Surgery”, Department of Surgery, Medical University of Vienna, Vienna, Austria

**Keywords:** medullary thyroid cancer, thyroid, calcitonin, calcium gluconate, pentagastrin

## Abstract

**Introduction:**

Pentagastrin (Pg) stimulated calcitonin (sCT) was used to enhance accuracy in medullary thyroid cancer (MTC) diagnosis. As it is now unavailable, calcium (Ca) has been recommended as an alternative. The aim of this study was to define gender-specific cut-off values to predict MTC in patients with elevated basal CT (bCT) following Pg-sCT and Ca-sCT stimulation and to compare the time courses of CT release during stimulation.

**Materials and methods:**

The stimulation tests were applied in 62 consecutive patients with thyroid nodules. Basal calcitonin was measured by chemiluminescent immunometric assay. All patients underwent thyroidectomy and bilateral central neck dissection. C-cell pathology was confirmed by histological and immunohistochemical evaluation.

**Results:**

In 39 (0.63) patients MTC was documented while isolated C-cell hyperplasia (CCH) was identified in 23 (0.37) patients. Medullary thyroid cancer was predicted in males with bCT values > 43 pg/mL or sCT concentrations > 470 pg/mL (Pg-sCT) or > 1500 pg/mL (Ca-sCT), and in females with bCT concentrations > 23 pg/mL or sCT concentrations > 200 pg/mL (Pg-sCT) or > 780 pg/mL (Ca-sCT), respectively. Pg-sCT correctly predicted MTC in 16 (0.66) compared to 13 (0.54) after Ca-sCT in males and in 12 (0.80) compared to 11 (0.73) in females; without statistical significance. In patients with CCH or low tumor burden, there was a tendency of faster CT release after Ca stimulation (CT peak after 3min in > 60%) compared to patients with advanced MTC (CT peak after 3min in < 10%).

**Conclusions:**

Using gender-specific cut-off values, Ca could replace Pg to predict MTC with similar diagnostic power.

## Introduction

Calcitonin (CT) concentrations reflect C-cell pathology in the preoperative work-up of thyroid nodules and, if elevated, serve to predict medullary thyroid cancer (MTC) at an early stage (*1*, *2*). Hypercalcitonemia, however, is not necessarily “thyroid C-cell-derived” (*3*). There are analytical, physiological, pharmacological and pathological factors that influence measured CT in patients without thyroid abnormalilties (*3*). Modern immunochemiluminometric assays are more sensitive and specific in terms of monomeric CT detection but these assays so far neither improved the discrimination between C-cell hyperplasia (CCH) and microMTC, nor facilitated the exclusion of interference by ectopic CT production of neuroendocrine tumors (*3*).

To increase the sensitivity of CT as a tumor marker, the application of CT stimulation tests has been recommended (*4*, *5*). These tests were described fifty years ago with the objective of early MTC diagnosis, even in asymptomatic patients with suspected hereditary C-cell disease who showed normal basal CT (bCT) concentrations (*6*, *7*). Thus, stimulation tests have been shown to be helpful in the diagnostic work-up of increased CT concentrations in questionable clinical circumstances but are even more important after surgical treatment of MTC to verify cure, recurrence or persistence (*8*, *9*).

Pentagastrin (Pg) has been considered as the best, most rapid, simplest and easiest applicable test for the early diagnosis of MTC. Numerous studies in the 1970s demonstrated that in comparison to Pg administration, the administration of calcium (Ca), either orally or *i.v.* produced slower and much smaller rises in CT (*10*-*15*). As a consequence Ca stimulation test was seldom applied and finally abandoned, as molecular genetic screening in families with multiple endocrine neoplasia (*16*) became more important in clinical practice than biochemical screening (*17*).

Calcitonin screening programs have been introduced into clinical practice mostly in European countries (*18*). Thereby, the early biochemical diagnosis of hereditary MTC has resulted in more favorable pathological stages of disease at the time of thyroidectomy than possible with clinical or radiological examinations alone (*19*). This has proved especially true when biochemical diagnoses were achieved by provocative testing in hereditary and sporadic disease (*2*). Pentagastrin stimulation became the “golden standard” in European countries. However, being unavailable in America and more recently also in Europe, Pg was to be replaced by other stimulants. A revival of the “short” Ca stimulation test (*11*) was introduced into clinical practice by Doyle *et al.* (*20*) who compared Pg-stimulated CT (Pg-sCT) and Ca-stimulated CT (Ca-sCT) concentrations for nonsmoking healthy adults without evidence of thyroid disorders to determine reference ranges of bCT as well as Pg-sCT and Ca-sCT concentrations.

As of 2013, the availability of Pg became less reliable and Pg was finally taken off the market at the end of 2015. Therefore, Ca stimulation should be introduced into clinical routine. As a consequence, all patients received both stimulation tests to ensure that results were comparable.

The aim of this study was to evaluate gender-specific bCT and sCT cut-off values and to retrospectively compare Pg-sCT and Ca-sCT concentrations in a precisely defined patient population with thyroid nodules for predicting MTC.

## Materials and methods

### Study design

As a consequence of a “CT screening program”, which was introduced into the routine work-up of nodular thyroid disease as a standard operating procedure (SOP) in 1994 (*21*), bCT concentrations are determined routinely in all patients visiting the outpatient departments that serve patients with nodular thyroid disease (General Hospital; University Hospital of Vienna - tertiary care center). Following the SOP, bCT is determined regardless of thyroid function, sonomorphology and/or size of the thyroid nodule(s).

The retrospective study comprised 62 consecutive patients including 39 (0.64) males (age: 57 years; absolute range: 43 - 70 years) and 23 (0.37) females (age: 54 years; absolute range: 40 - 68 years) with nodular thyroid disease, normal thyroid function tests and reproducible elevated bCT who were sent for initial surgical treatment during the years 2014 and 2015.

All patients had elevated fasting bCT concentrations upon initial evaluation (normal range: male < 8 pg/mL; female < 6 pg/mL) and were assigned to further examinations in a close interval of one week: first Pg-sCT and second Ca-sCT test, as described below. In all patients, stimulated sCT concentrations were at least twice higher than the bCT concentrations and/or > 100 pg/mL in one of the two stimulation tests.

All possible causes (such as restricted kidney function, intake of proton pump inhibitors, *etc*.) known to modify bCT concentrations were excluded before applying CT screening. No patient was a member of a known MTC family or had undergone surgery for MTC.

The study was approved by the local Ethics Committee (EK1506/2014) of the Medical University of Vienna and adhered to the Declaration of Helsinki. Informed consent was obtained from each patient for all diagnostic and therapeutic procedures.

### Methods

According to the time protocol described below for the Pg and Ca stimulation tests, about 3 to 5 mL fasting blood samples were collected at each time point in Vacuette® separation gel tubes (Greiner Group, Kremsmünster, Austria) without additives. The stimulation tests were performed no later than 9 o’clock in the morning.

During transport to the laboratory, and within one hour, blood samples clotted and were subsequently administrated and centrifuged at 2000xg for 10 minutes at 4 °C.

The obtained serum samples were stored in a refrigerator at 6 °C until analyses (on the same or the next day). In the case of weekends or holidays, samples were frozen at -20 °C and thawed at room temperature in a water bath for subsequent analyses. These procedures facilitated neglecting loss of CT immunoreactivity.

Calcitonin concentrations were determined with a chemiluminescent immunometric assay from Diagnostic Products Corporation (DPC, Los Angeles, CA, USA), running as a fully automated test on a Immulite 2000 Immunoassay System (Siemens Health Care), as described previously (*4*). Assays were calibrated by two-point calibrators, suggesting a linear dose-response relationship within the range of 2 to 2000 pg/mL. Sample concentrations exceeding the upper reporting range were diluted with Multi-Diluent 2 from DPC. In the latter case, stimulation tests are an appropriate measure to detect obscure results, as well as observations on deviations from linear dilution behavior. There was no cross-reactivity of the assay against 12 peptide hormones including prepro-CT. Day-to-day imprecision was evaluated by control samples purchased from DPC with mean concentrations of 10 pg/mL and 210 pg/mL CT, resulting in coefficients of variations of 10% and 7%, respectively. Additionally, the laboratory successfully participated in CT external quality trials of Instand e.V. (Germany) and the Royal College of Pathologists of Australasia Quality Assurance Program (Australia).

All patients were stimulated by Pg and Ca after at least 6 hours of fasting before the test. The median interval between the two tests was 7 days.

Blood samples for CT determination were drawn with an indwelling catheter before and 2, 3, 5 and 10 minutes after an *i.v.* bolus of 0.5 µg/kg Pg (Cambridge Laboratories Limited; Newcastle upon Tyne, UK) or after a 30-second infusion of 2.5 mg Ca *per* kg body weight (Ca gluconate 10% [10 mL containing Ca gluconate 2.25 mmol = 90 mg Ca^2+^; Calcium Braun 10%®, B. Braun Melsungen AG, Melsungen, Germany]).

All patients underwent primary (total) thyroidectomy and bilateral central neck dissection (= level 6 [extirpation of the prelaryngeal and pretracheal lymph nodes including bilateral microdissection of the lymphatic tissue along both recurrent laryngeal nerves]) irrespective of preoperative bCT and sCT concentrations. In selected patients, surgery was completed by (“functional”) microdissection of both lateral neck compartments (levels 2 to 5). No permanent complications (hypoparathyroidism, paralysis of the recurrent nerve) were observed in the study patients.

All thyroid glands were submitted to pathology and inspected macroscopically. The entire organ was sectioned in slices of approximately three to five millimeters and frozen sections were performed of the macroscopically identified primary tumor. The entire remaining thyroid gland was serially blocked in paraffin. Sections of each block, as well as three sections of each submitted lymph node, were stained with hematoxylin and eosin. Immunohistochemistry was performed using the avidin-biotin-peroxidase technique. A section of each block was immunostained for CT using an available antibody (Chemicon, Temecula, USA) in a dilution of 1:600.

C-cell hyperplasia was diagnosed when at least one area with > 50 C-cells *per* one low-power field (x 100) was identified in both thyroid lobes. C-cell hyperplasia was morphologically classified as focal, diffuse, nodular (summarized as “physiological”) or “neoplastic” CCH. Medullary thyroid cancer was diagnosed if a focal loss or reduplication of the basement membrane was observed by immunohistochemistry. The tumors were classified in accordance with the American Joint Committee on Cancer Staging Manual of 2010 (*22*).

Possible germline mutations were investigated routinely in all study patients by screening exons 5, 8, 10, 11, 13, 14, 15 and 16 of the Rearranged during Transfection (RET) proto-oncogene using high-resolution melting real-time PCR (*23*).

All biochemical data and histological findings in the thyroid gland and in dissected lymph nodes were collected prospectively and correlated retrospectively.

### Statistical analysis

The highest CT concentrations after stimulation (either after 2, 3, 5 or 10 minutes) were used for further statistical analysis in all comparisions. Increase in CT after stimulation was calculated as maximum CT concentration during stimulation minus bCT concentration (= ∆ value). Normal distribution was assessed with the Shapiro-Wilk test and visual inspection of histograms. As the analysis showed skewed distribution in all relevant parameters, data are presented as median and interquartile range (IQR, 25th percentile - 75th percentile). Spearman’s rank-order correlations were run to assess the relationship between Ca- and Pg-Stimulation and between bCT and sCT after both types of stimulation. A Wilcoxon signed-rank test was used for any group comparison of continuous parameters. Fisher’s exact test was used to compare binominal proportions. P values < 0.05 were considered significant.

In order to define cut-off values, receivers operating characteristic (ROC) curves were calculated and described using the area under the curve (AUC), confidence intervals (CI), and P values. In view of gender-specific differences, sensitivity and specificity as calculated by the ROC curves were used to establish cut-off values for the discrimination between CCH and MTC for both female and male patients (*2*, *24*). All calculations were done with SPSS Statistics (released 2015, IBM SPSS Statistics for Windows, Armonk, NY: IBM Corp) version 23.0 for Windows and Microsoft Excel 2016 for Windows.

## Results

Medullary thyroid cancer was documented in 24/39 (0.62) males and 15/23 (0.65) females, while isolated CCH (“CCH only”) was identified in 15/39 (0.38) and 8/39 (0.35), respectively. Table 1 summarizes further pathological and genetic details including the tumor classifications according to International Union Against Cancer (UICC) 2010 (*22*).

**Table 1:** Morphological findings and tumor classification of patients investigated

	**Males (N = 39)**	**Females (N = 23)**
Isolated CCH, N (proportion)	15 (0.38)	8 (0.35)
Neoplastic	8 (0.53)	4 (0.5)
Physiological (diffuse or nodular)	7 (0.47)	4 (0.5)
MTC, N (proportion)^ *^	24 (0.62)	15 (0.65)
pT1a	14 (0.58)	9 (0.60)
pT1b	4 (0.17)	4 (0.27)
pT2	2 (0.08)	2 (0.13)
pT3	4 (0.17)	0
pT4	0	0
Multifocality, N (proportion)	5 (0.21)	1 (0.07)
Tumor diameter, (mm)	6 (1 - 19) (absolute range: 1 - 87)	9 (4 - 15) (absolute range: 1 - 21)
Concomitant CCH, N (proportion)	18 (0.75)	5 (0.33)
Neoplastic	12 (0.67)	3 (0.60)
Physiological	6 (0.33)	2(0.40)
Meta, N (proportion)	8 (0.33)	3 (0.20)
N1a	2 (0.25)	2 (0.67)
N1b	6 (0.75)	1 (0.33)
Dist Meta, N (proportion)	3 (0.13) liver; N = 2 liver + lung + bone; N = 2	0
RET mutation^, ^N (proportion)	1 (0.04) exon 13, codon 791; N = 1	4 (0.27) exon 13/codon 791; N = 1 exon 13/768; N = 3
CCH - C-cell hyperplasia. MTC - medullary thyroid cancer. ^*^Local tumor stage according to UICC 2010 - pT1a ≤ 10 mm; pT1b = 11 - 20 mm; pT2 = 21 - 40 mm; pT3 > 40 mm or any size with with extrathyroidal extension; pT4 = moderately advanced or very advanced disease. Meta - lymph node metastasis: N1a - central lymph node metastasis, N1b - lateral lymph node metastasis. Dist Meta - distant metastasis. RET mutation - mutation in the rearranged during transfection (RET) proto-oncogene, found after diagnosis of MTC.		

After stimulation with Pg or Ca, all patients showed at least two-fold increase in maximum sCT compared to the bCT concentrations (Ca stimulation: 738 pg/mL [IQR: 372 - 3563], Pg stimulation: 275 pg/mL [IQR: 105 - 3008], basal: 25 pg/mL [IQR: 13 - 150]). Regardless of gender, the median of maximum sCT release was higher with Ca than with Pg stimulation (P < 0.001).

**Figure 1:**
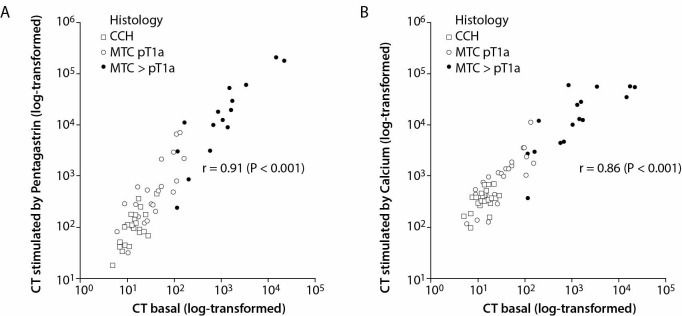
Plot of basal calcitonin *vs.* pentagastrin-stimulated calcitonin (A) and calcium-stimulated calcitonin (B) for patients with thyroid nodules. CT - calcitonin. CCH - C-cell hyperplasia. MTC pT1a - medullary thyroid cancer stage pT1a (≤ 10 mm). MTC > pT1a - medullary thyroid cancer higher stage than pT1a (> 11 mm).

There was a strong correlation between the maximum Pg-sCT and Ca-sCT, respectively (r = 0.90; P < 0.001). Similarly, a strong and significant correlation between bCT and Pg-sCT and Ca-sCT was observed (r = 0.91 and r = 0.86; both P < 0.001). There was a considerable overlap of CT concentrations between CCH and MTC classified as pT1a (tumor diameter ≤ 10 mm), irrespective of the stimulation test. Higher stimulations were observed in MTC classified as pT1b or higher (tumor diameter ≥ 11 mm; Figure 1).

### Gender-specific cut-off values

The AUCs for the ROC curves of bCT, Pg-sCT, Ca-sCT and the absolute increase after each of the two stimulation tests (= ∆ values) are shown in Table 2. As the AUCs of bCT and both Pg and Ca stimulation tests were high (> 0.80), with narrow CI reaching the level of significance, all tests seemed useful to discriminate between CCH and MTC. The AUC of the Δ values for both stimulation techniques were very close to the AUC of the maximum sCT. Therefore, Δ values were excluded from further analysis.

**Table 2:** ROC curves for the discrimination between C-cell hyperplasia and medullary thyroid cancer

	**Males**			**Females**		
	**AUC**	**CI**	**P**	**AUC**	**CI**	**P**
**bCT**	0.87	0.75 - 0.99	< 0.001	0.90	0.77 - 1.00	0.002
**Pg-sCT**	0.88	0.77 - 0.99	< 0.001	0.94	0.85 - 1.00	< 0.001
**Ca-sCT**	0.85	0.72 - 0.98	< 0.001	0.88	0.73 - 1.00	0.004
**Pg - ∆values**	0.88	0.76 - 0.99	< 0.001	0.93	0.82 - 1.00	< 0.001
**Ca - ∆values**	0.85	0.72 - 0.98	< 0.001	0.86	0.71 - 1.00	0.006
AUC - area under the curve. CI - confidence interval. bCT - basal calcitonin value. Pg-sCT - pentagastrin stimulated calcitonin value. Ca-sCT - calcitonin stimulated calcitonin value. Pg - ∆ values - pentagastin-differences between peak stimulated calcitonin and basal calcitonin. Ca - ∆ values - calcium-differences between peak stimulated calcitonin and basal calcitonin.						

**Figure 2:**
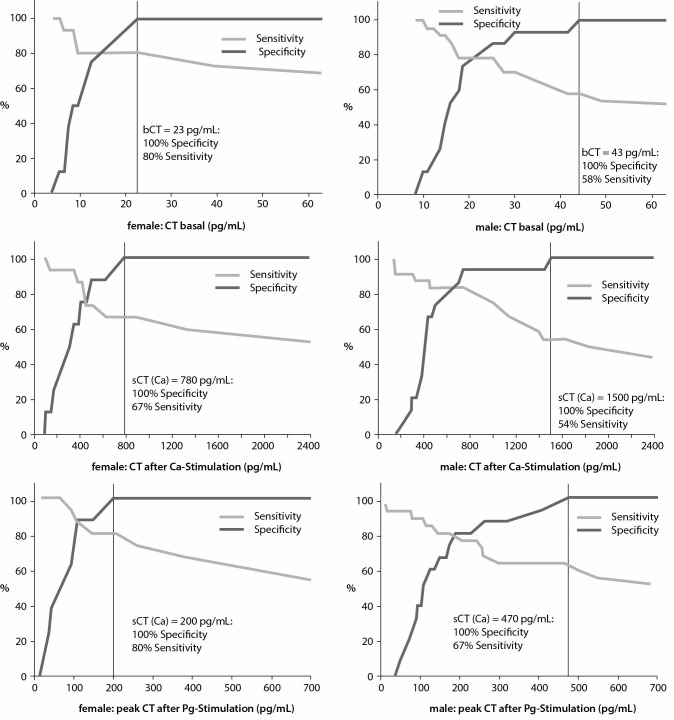
Plots of basal calcitonin (bCT), pentagastrin-stimulated calcitonin (sCT(Pg)) and calcium-stimulated calcitonin (sCT(Ca)) values against specificity (dark gray) and sensitivity (light gray) for visual determination of optimal cut-off values in female and male patients with thyroid nodules to differentiate between C-cell hyperplasia and medullary thyroid cancer.

Figure 2 shows the CT concentrations *versus* sensitivity and specificity calculated by ROC curves. A relevant overlap of CCH and MTC in patients with mildly elevated CT levels was documented in all tests and in both gender groups. Medullary thyroid cancer was predicted in 100% of the male patients with bCT concentrations > 43 pg/mL (sensitivity: 58%) or Pg-sCT > 470 pg/mL (sensitivity: 67%) and Ca-sCT > 1500 pg/mL (sensitivity: 54%) and in 100% of the female subjects with bCT concentrations > 23 pg/mL (sensitivity: 80%) or Pg-sCT concentrations > 200 pg/mL (sensitivity: 80%) and Ca-sCT concentrations > 780 pg/mL (sensitivity: 67%), respectively.

The bCT and sCT concentrations were combined in order to optimize the prediction of C-cell morphology and thus MTC diagnosis.

Patients were grouped as follows: in Group 1, bCT and sCT were below the cut-off values, and therefore CCH or MTC could not be definitively predicted by either bCT or sCT. In Group 2, MTC was predicted in all patients either by bCT and/or sCT level by exceeding the cut-off values defined above (Tables 3a and 3b).

**Table 3:** Prediction of C-cell hyperplasia and medullary thyroid cancer in males and females

**Male patients (N = 39)**																	
**Stimulation** **by**	**Group**	**Prediction ** **of morphology**		**N (proportion)**	**Combination of**			**Definitive diagnosis**	**N (proportion)**	pT1a^*^	pT1b^*^	pT2^*^	pT3^*^	pT4^*^		**N1**	**M1**
					**bCT** **(pg/mL)**	**sCT** **(pg/mL)**				**N (proportion)**							
Pg	1	CCH or MTC		23 (0.59)	≥ 8 ≤ 43	≤ 470		CCH	15 (0.65)								
								MTC	8 (0.35)	8 (1.0)							
	2	MTC		16 (0.41)	≥ 8 ≤ 43	> 470		MTC	16 (1.0)	6 (0.38)	4 (0.25)	2 (0.13)	4 (0.25)		0	5 (0.31)	3 (0.19)
					> 43	≤ 470											
Ca	2	MTC		13 (0.33)	≥ 8 ≤ 43	> 1500		MTC	13 (1.0)	3 (0.23)	4 (0.31)	2 (0.15)	4 (0.31)		0	5 (0.38)	3 (0.23)
					> 43	≤ 1500											
	1	CCH or MTC		26 (0.67)	≥ 8 ≤ 43	≤ 1500		MTC	11 (0.42)	11 (1.0)							
								CCH	15 (0.58)								
**Female patients (N = 23)**																	
Pg	1	CCH or MTC	11 (0.48)		≥ 6 ≤ 24		≤ 200	CCH	8 (0.73)								
								MTC	3 (0.27)	3 (1.0)						1 (0.33)	
	2	MTC	12 (0.52)		≥ 6 ≤ 24		> 200	MTC	12 (1.0)	6 (0.50)	4 (0.33)	2 (0.17)	0		0	2 (0.17)	
					> 24		≤ 200										
Ca	2	MTC	11 (0.48)		≥ 6 ≤ 24		> 780	MTC	11 (1.0)	5 (0.45)	4 (0.36)	2 (0.18)	0		0	2 (0.18)	
					> 24		≤ 780										
	1	CCH or MTC	12 (0.52)		≥ 6 ≤ 24		≤ 780	MTC	4 (0.33)	4 (1.0)						1 (0.09)	
								CCH	8 (0.66)								
bCT - basal calcitonin. sCT - stimulated calcitonin. ^*^Local tumor classification according to UICC 2010 - pT1a ≤ 10mm; pT1b = 11 - 20 mm; pT2 = 21- 40 mm; pT3 > 40 mm or any size with with extrathyroidal extension; pT4 = moderately advanced or very advanced disease. N1 - lymph node metastasis. M1 - distant metastasis. Pg - pentagastrin. Ca - calcium. Group 1 - C-cell disease not predictable (CCH or MTC possible). Group 2 - MTC predicted in all patients. CCH - C-cell hyperplasia, MTC - medullary thyroid cancer. Gray zone represents correctly predicted MTC with pentagastrin stimulation in 16 (0.67) of 24 finally diagnosed MTCs compared to 13 (0.54) after calcium stimulation (Fisher’s exact test; P = 0.556) for male patients, and in 12 (0.80) of 15 finally diagnosed MTCs compared to 11 (0.73) patients after calcium stimulation (Fisher’s exact test: P = 1.000) for female patients.																	

#### Male patients

##### Pg stimulation

Group 1 (CCH or MTC): Considering low bCT (≥ 8 to ≤ 43 pg/mL) and sCT (≤ 470 pg/mL) concentrations in 23/39 (0.59) males, no clear biochemical differentiation between CCH and MTC was possible. MTC was identified in 15/23 (0.65) and CCH in 8/23 (0.35) males, respectively. All tumors were classified as pT1a (tumor size: ≤ 10 mm). None had lymph node or distant metastasis (Table 3).

Group 2 (MTC only): MTC was predicted in 16/16 males with a bCT concentration > 43 pg/mL or, regardless of bCT concentrations, with a sCT concentration > 470 pg/mL. In all 16 patients, the malignancy was definitively confirmed histologically. Five patients with lymph node and three with distant metastasis were documented (Table 3).

##### Ca stimulation

Group 1 (CCH or MTC): Similarly, no clear discrimination between CCH and MTC was achieved by Ca stimulation (sCT ≤ 1500 pg/mL) in 26/39 (0.67) males. This group consisted of 15/26 (0.58) patients with CCH and 11/26 (0.28) patients with MTC, all with pT1a tumors (Table 3).

Group 2 (MTC only): MTC was identified in 13/13 males with a bCT concentration > 43 pg/mL or, regardless of bCT concentration, with a sCT concentration > 1500 pg/mL after Ca stimulation. Five patients with lymph node and three with distant metastasis were documented (Table 3).

Finally, MTC was diagnosed in 24/39 (0.61) males. Pg stimulation correctly predicted MTC in 16/24 (0.67) and Ca stimulation in 13/24 (0.54) males, respectively. Pg stimulation predicted MTC in three more patients compared to Ca, yet without statistical significance (P = 0.556).

#### Female patients

##### Pg stimulation

Group 1 (MTC or CCH): Considering bCT (≥ 6 to ≤ 24 pg/mL) and sCT (≤ 200 pg/mL) concentrations in 11/23 (0.48) females, no clear biochemical differentiation between CCH and MTC was achieved, as 8/11 (0.73) females suffered from CCH and 3/11 (0.27) from MTC. All malignant tumors were classified as pT1a. Bilateral central neck dissection revealed unilateral central lymph node metastasis in one female patient (Table 3).

Group 2 (MTC only): MTC was predicted in 12/12 females with a bCT concentration > 24 pg/mL or, regardless of bCT concentrations, with a sCT concentration > 200 pg/mL after Pg stimulation. Two patients with lymph node metastases were documented (Table 3).

##### Ca stimulation

Group 1 (MTC or CCH): Discrimination between CCH and MTC failed after Ca stimulation (sCT ≤ 780 pg/mL) in 12/23 (0.52) females. This group comprised 8/12 (0.67) patients with CCH and 4/12 (0.33) patients with MTC, all presenting with pT1a tumors. Bilateral central neck dissection revealed unilateral central lymph node metastasis in one female subject (Table 3b).

Group 2 (MTC only): MTC was identified in 11/11 females with a bCT concentration > 24 pg/mL or, regardless of bCT concentrations, with a sCT concentration > 780 pg/mL after Ca stimulation. The pTNM classification is summarized in Table 3b.

MTC was finally diagnosed in 15/23 (0.65) female patients. Pg stimulation predicted MTC in 12/15 (0.80) and Ca stimulation in 11/15 (0.73) patients. Pg stimulation predicted two more patients than Ca stimulation, yet without statistical significance (P = 1.000).

**Figure 3:**
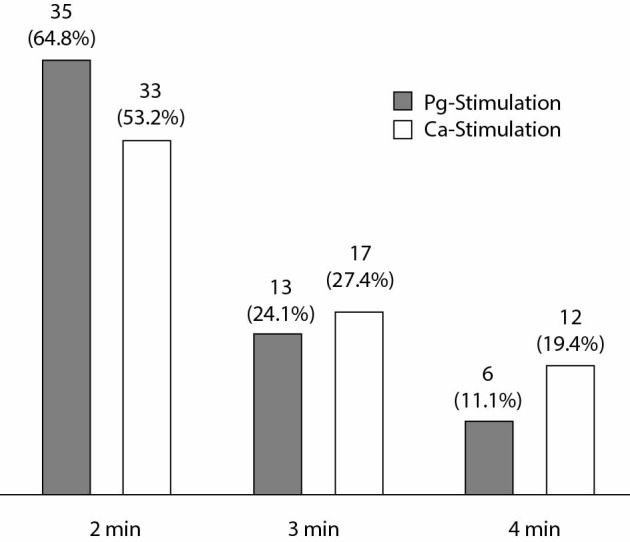
Time (minutes after injection) of calcitonin peak value after pentagastrin or calcium stimulation in patients with thyroid nodules. Results are given as absolute number and percentage of all patients (%).

The time courses of sCT peak release after Pg and Ca stimulation are summarized in Figure 3. Calcitonin peak values were reached 2 minutes after stimulation in more than 50%, after 3 minutes in around 25%, and after 5 minutes in 10 to 20% of patients. In both tests and in all patients, sCT after 10 minutes was either lower or similar to sCT after 5 minutes. Thus, sCT concentrations after 10 minutes were not considered.

The occurence of sCT peak in minutes after Ca stimulation are given in Table 4. The patients were subclassified by C-cell pathology and gender. In females, there was a tendency towards earlier peak sCT in CCH than in MTC. This tendency was not found among male patients. Table 5 summarizes the occurence of Ca-sCT peak according to CCH and MTC tumor classification, respectively. Fast maximal sCT release within 2 minutes after stimulation was observed in CCH and in about two thirds of pT1a tumors. In all other MTCs (pT1b to pT3), peak sCT mostly occurred later. In lymph node-negative MTC patients, peak sCT concentrations frequently appeared within 2 to 3 minutes, whereas in subjects with lymph node metastasis (N1), peak sCT was reached later.

**Table 4:** Time of calcitonin peak value after calcium stimulation in patients with thyroid nodules

	**MTC**		**CCH**	
**Minutes** **after stimulation**	**male**	**female**	**male**	**female**
	N (proportion)	N (proportion)	N (proportion)	N (proportion)
**2**	12 (0.50)	7 (0.47)	8 (0.54)	6 (0.75)
**3**	5 (0.21)	5 (0.33)	5 (0.33)	2 (0.25)
**5**	7 (0.29)	3 (0.20)	2 (0.13)	0 (0.0)
MTC - medullary thyroid cancer. CCH - C-cell hyperplasia.				

**Table 5:** Time of calcitonin peak value after calcium stimulation in patients with thyroid nodules subdivided by tumor classification and lymph node involvement

	**CCH**	**MTC**					
**Minutes** **after stimulation**	**pT1a**	**pT1b**	**pT2**	**pT3**	**N0**	**N1**
	N (proportion)	N (proportion)	N (proportion)	N (proportion)	N (proportion)	N (proportion)	N (proportion)
**2**	14 (0.61)	16 (0.70)	2 (0.25)	1 (0.25)	0	18 (0.64)	1 (0.9)
**3**	7 (0.30)	4 (0.17)	4 (0.50)	1 (0.25)	1 (0.25)	7 (0.25)	3 (0.27)
**5**	2 (0.9)	3 (0.13)	2 (0.25)	2 (0.50)	3 (0.75)	3 (0.11)	7 (0.64)
CCH - C-cell hyperplasia. MTC - medullary thyroid cancer. Local tumor stage according to UICC 2010 - pT1a ≤ 10 mm; pT1b = 11 - 20 mm; pT2 = 21 - 40 mm; pT3 > 40 mm or any size with with extrathyroidal extension; pT4 = moderately advanced or very advanced disease. N0 - no lymph node metastasis present. N1 - lymph node metastasis.							

## Discussion

To our knowledge, this is currently the largest study based on a uniform and rigorously followed diagnostic and surgical protocol analyzing a representative group of patients with CCH and MTC. The objective was to relate bCT and maximal sCT concentrations with pathohistological and immunhistochemical findings by testing CT release in one and the same patient with the two different stimulation agents and to precisely define relevant cut-off values for Ca-sCT. The results of this study suggest a similar diagnostic value of sCT by Ca and Pg. Irrespective of the stimulation agent, all patients showed an increase in sCT at least twice higher than bCT concentrations. There was a strong linear correlation between maximum CT releases stimulated either by Pg or Ca. There are specific bCT and sCT values in males and females. When CT exceeds the gender-dependent cut-off of 100% specificity for diagnosis, MTC can definitively be predicted (100% positive predictive value) and “adequate” (radical) surgical strategies are subsequently to be applied in these patients (*1*, *2*). Patients below these cut-off values have either CCH or MTC pT1a. Neither Pg-sCT nor Ca-sCT was seen to be helpful in definitively ruling out MTC in these patients. The overlap of patients with CCH or early-stage MTC (pT1a) was nearly identical after applying both tests. These patients are eligible for less radical surgery comprising (total) thyroidectomy with bilateral central (level 6) neck dissection (*2*).

Only one similar report in the literature has compared Pg-sCT and Ca-sCT (*25*). Colombo *et al.* mainly focused on initial clinical experience with Ca stimulation in patients with suspected C-cell disease (*25*). However, the authors followed different surgical protocols and in fact only 12 patients received both Ca and Pg stimulations to compare and evaluate the stimulation tests for clinical practatice. Kowalsky *et al.* have recently suggested a similar diagnostic value of Ca gluconate compared to Pg during follow-up after thyroidectomy (*26*).

Although using the same two-site chemiluminescent immunometric CT assay, some differences were identified in the cut-off values after Ca stimulation compared to the paper published by Colombo *et al.* (*25*). This may be due to modifying the recommended protocol by Doyle *et al.*: blood sampling was extended by an additional sampling 3 minutes after either Pg or Ca application (*20*). Unpublished preliminary data by one of the authors (G.A.) have documented higher sCT levels after 3 minutes compared to 2 minutes in some patients. The 3-minutes sampling may allow a more precise interpretation in tumors which release the highest sCT after 3 minutes. The sCT concentrations 10 minutes after stimulation failed to provide any additional information and may be omitted, thus shortening the test. Adding 3-minutes sampling may be also relevant for the indication and extent of surgery. Regarding the time course, there was a tendency towards earlier peak sCT for CCH and lymph node-negative low-tumor classes compared to more advanced MTC. In the assessment of Ca stimulation with concentrations of bCT and sCT below the gender-specific cut-off values, an occurrence of early-peak sCT will increase the predictive probability of CCH or low tumor burden.

In our experience, both stimulation tests are generally well tolerated. According to the patients’ clinical records, there were no severe adverse events whatsoever (*27*).

Striking controversies still maintain with regard to the use of routine CT measurement in patients with nodular thyroid disease (*28*-*30*). However, if bCT concentrations are determined to be repeatedly borderline-elevated, a stimulation test is strongly recommended to enhance diagnostic accuracy (*31*).

The limitation of this study may be the small sample size for the determination of an “absolute” cut-off value. However, MTC is an “orphan disease”. Therefore, the numbers of patients suffering from MTC are limited even in tertiary care centers. On the other hand, the advantage of this study is the uniform diagnostic and surgical protocol applied. This seems the only reasonable basis to define “optimal” cut-off concentrations after standardized Ca stimulation.

In conclusion, the high-dose short Ca stimulation test based on novel, gender-specific cut-off values is a potent tool that can be applied widely and at low costs, while providing additional clinical information in selected patients with (mildly) elevated bCT to differentiate between various C-cell diseases. If a stimulation test is indicated, Ca may replace Pg as the “new standard” with equivalently high diagnostic quality to predict MTC.
